# The effect of A1298c polymorphism of the MTHFR gene on anti‐Müllerian hormone levels: experimental and Web‐based analysis

**DOI:** 10.1002/jcla.23948

**Published:** 2021-08-08

**Authors:** Seyedeh Zahra Shahrokhi, Faranak Kazerouni, Firouzeh Ghaffari, Morteza Hadizadeh, Zahra Zolfaghary

**Affiliations:** ^1^ Department of Laboratory Medicine School of Allied Medical Sciences Shahid Beheshti University of Medical Sciences Tehran Iran; ^2^ Department of Endocrinology and Female Infertility Reproductive Biomedicine Research Center Royan Institute for Reproductive Biomedicine ACECR Tehran Iran; ^3^ Physiology Research Center Institute of Basic and Clinical Physiology Sciences Kerman University of Medical Sciences Kerman Iran; ^4^ Reproductive Epidemiology Research Center Royan Institute for Reproductive Biomedicine ACECR Tehran Iran

**Keywords:** anti‐müllerian hormone, male factor infertility, MTHFR A1298C polymorphism, oocyte retrieved, PCR‐RFLP

## Abstract

**Background:**

The 5,10‐methylenetetrahydrofolate reductase (MTHFR) is an important enzyme of folate and methionine metabolism, which is expressed in human oocytes and preimplantation. Due to the involvement of MTHFR *in* female reproduction, *we* tend to evaluate the influence of MTHFR A1298C polymorphism on ovarian marker reserves such as serum anti‐Müllerian hormone (AMH) levels in women after in vitro fertilization (IVF)/intracytoplasmic sperm injection (ICSI).

**Methods:**

A total of 100 women, who underwent ART treatment due to male factor infertility, were recruited into this study. MTHFR A1298C polymorphism was detected by polymerase chain reaction‐restriction fragment length polymorphism (PCR‐RFLP) technique, and serum AMH concentrations were measured by an ultrasensitive enzyme‐linked immunosorbent assay (ELISA).

**Results:**

Women with the CC genotype had higher AMH levels (4.15 ± 1.67 ng/ml), albeit not significant, than carriers with other genotypes after ovarian stimulation. No significant differences existed in terms of miscarriage and live birth rates among different genotype groups.

**Conclusion:**

The presence of the C mutant allele of the 1298 polymorphism in the MTHFR gene led to an increasing trend in serum AMH concentrations; however, the numbers of oocytes retrieved decreased in women with mutated genotypes. The influence of the MTHFR C677T polymorphism on embryo quality and pregnancy rate after ART cycles remains unclear.

## Graphical abstract

Schematic figure indicates the important role of the MTHFR enzyme in folate metabolism. MTHFR, methylenetetrahydrofolate reductase; SAH, S‐adenosylhomocysteine; SAM, S‐adenosylmethionine; THF, tetrahydrofolate.

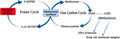

## 1 INTRODUCTION

In assisted reproductive technology (ART), high variability within the ovarian response to gonadotropins represents one of the most intractable issues, ranging from hypo‐response to hyper‐response, which results in cycle cancelation or associated with ovarian hyperstimulation syndrome.^1^ Therefore, the identification of accurate predictors of ovarian response and optimization stimulation protocols could be a crucial step for the successful outcome of ART.^2^ Despite significant improvements in infertility treatment, the individual ovarian response to The FSH is unrespectable and may depend on their genetic makeup.^3^, ^4^ Maybe, the patient genetic background better than the stimulation protocol determined the ovarian response.

The flavoenzyme 5,10‐methylenetetrahydrofolate reductase (MTHFR) is a key regulatory enzyme in folate metabolism, which involved in the process of DNA synthesis and regulation of homocysteine levels.^5^ It catalyzes the conversion of 5,10‐methylenetetrahydrofolate to 5‐methyltetrahydrofolate as a methyl group donor in homocysteine remethylation to methionine.^5^ A common A>C mutation in the MTHFR gene at nucleotide position 1298 causes the substitution of glutamate to alanine in the MTHFR protein and a consequent reduction in enzyme activity, approximately 40% of the wild‐type individuals.^6^ Although decreased MTHFR activity due to A1298C polymorphism may lead to impaired DNA methylation, which is essential for normal embryo development,^7^ only a few studies have evaluated the relationship between the MTHFR A1298C polymorphism and infertility, even with controversial results.^8^, ^9^, ^10^, ^11^ We have reported that women carrying homozygous (1298CC) genotype were less likely to achieve pregnancy and deliver a baby after IVF than those with the wild‐type (1298AA) genotype. However, a recent study failed to find an association between A1298C polymorphism with embryo quality, ongoing pregnancy rate, and abortion rate.^9^

The MTHFR gene expression was observed in human oocytes and preimplantation embryos.^9^ It has been suggested that it is involved in many physiologic processes such as folliculogenesis and maintenance of genomic stability in oocytes.^12^ During folliculogenesis, in which granulosa cells (GC) proliferate in response to FSH, the ovarian response of patients to FSH stimulation could be related to MTHFR polymorphisms.^13^, ^14^ Due to the important role of MTHFR in female reproduction, we tend to analyze the MTHFR A1298C polymorphisms in 100 women who underwent ART treatment to determine their relationship with an ovarian reserve and ovarian stimulation.

## 2 MATERIALS AND METHODS

### 2.1 Patient population

This study was conducted at Royan Institute from April 2018 to March 2019. Informed consent was obtained from all participants prior to their participation in the study, and the protocol was approved by the Local Ethics Committee of the Shahid Beheshti University of Medical Sciences (reference no IR.SBMU.RETECH.REC.1397.293). The investigated population included 100 women with a normal ovarian function who referred to infertility clinic due to male factor infertility. Further selection criteria for this study were as follows: 20–37 years of age, normal menstrual cycle (length: 25–35 days), no evidence of endocrine disorders, and no recent hormone therapy (within the 3 months). The subjects with a history of ovarian surgery and endocrine abnormalities, such as polycystic ovary syndrome (PCOS) and endometriosis, were excluded from the study.

### 2.2 Blood sampling

Fresh blood samples were collected during the 2nd or 3rd day of the menstrual cycle for AMH measurement and genomic DNA extraction. In order to measure the AMH levels, samples were centrifuged at 2,000 g for 10 min, and all the sera were stored at −80°C. The AMH levels in serum were measured using an ultrasensitive enzyme‐linked immunosorbent assay (ELISA) kit (cat. AL‐105‐i, Ansh Laboratories), with limits of detection set at 0.023 ng/mL.

### 2.3 Genotyping

Genomic DNA was extracted from leukocytes by the QIAamp DNA Blood Mini Kit.

(Qiagen) according to the manufacturer's protocol. For determination of the MTHFR gene A1298C genotype, polymerase chain reaction‐restriction fragment length polymorphism (PCR‐RFLP) technique was performed using forward 5'‐ATGTGGGGGGAGGAGCTGAC‐3’ and reverse 5'‐GTCTCCCAACTTACCCTTCTCCC‐3’ primers. PCR amplification is carried out in 25 µl total reaction volume containing template DNA (3 µl), 0.5 ml of each of the primers, MgCl2 (1 µl), dNTP (0.25 µM), ddH2O (17 µl), 10 × PCR buffer (2.5 µl), and Taq polymerase (0.25 µl). The PCR cycling conditions were as follows: initial denaturation for 5 min at 95°C, 30 cycles of denaturation for 30 s at 95°C, annealing for 30 s at 58°C, elongation for 35 s at 72°C, and a final extension for 10 min at 72°C. The 163‐bp PCR product was digested by the restriction enzyme MboII (Fermentas) at 37°C overnight and then was separated on SyberSafe‐stained 4% agarose gel electrophoresis. The AA genotype (wild) produced 5 fragments (56, 31, 30, 28, and 18 bp size), whereas the CC genotype (homozygote) produced 4 fragments (84, 31, 30, and 18 bp), and also, the AC genotype (heterozygote) yields 6 fragments (84, 56, 31, 30, 28, and 18 bp).

### 2.4 Treatment protocol

Patients were treated with GnRH agonist and antagonist protocol. A detailed description of the stimulation protocols, follicle monitoring, hCG triggering, oocyte retrieval, and embryo transfer has been described.^15^ Primary endpoints included the total number of retrieved oocytes and the determination of the effect of the MTHFR A1298C polymorphism on serum AMH levels. Clinical pregnancy, miscarriage, and live birth rates were considered as secondary endpoints.^16^ A poor ovarian response was defined as ˂4 oocytes retrieved, a normal ovarian response as 4 to 15 oocytes retrieved, and a high ovarian response as ˃ 15 oocytes retrieved.^17^

### 2.5 In silico analysis

The DNA and amino acid sequences of the MTHFR gene (with AC: AY338232.1 and P42898) were obtained from NCBI (http://www.ncbi.nlm.nih.gov/) and UniProt (https://www.uniprot.org/), respectively. Amino acid sequences were analyzed by UniProt, ProtParam (https://web.expasy.org/protparam/), and Biosynthesis (https://www.biosyn.com/peptidepropertycalculatorlanding.aspx). The effect of Glu429Ala substitution on protein function was evaluated by SNAP2 (screening for non‐acceptable polymorphisms; https://rostlab.org/services/snap/), PolyPhen‐2 (http://genetics.bwh.harvard.edu/pph2/), and SIFT (scale‐invariant feature transform; http://sift.jcvi.org/) databases. The effect of A1286C substitution on the mRNA structure was determined by RNAsnp database (http://rth.dk/resources/rnasnp/).

### 2.6 Statistical analysis

All statistical analyses were carried out with SPSS software version 20 (IBM Corporation). Parametric variables were compared by one‐way ANOVA test, and nonparametric variables, by the Kruskal‐Wallis test, and data were presented as the mean ± standard deviation (SD) or number and percent. To test for associations between genotypes and categorical variables, the chi‐squared test was used. Distributions of the genotypes were checked with a Hardy‐Weinberg equilibrium test. In order to predict efficacy factors oocyte retrieve, we used linear regression. Multiple logistic regression analyses were used to indicate the association between the dependent (pregnancy compared with no pregnancy) and independent variables. Adjusted odds ratios (aORs) and 95% confidence interval (CIs) were reported. *p*‐values of <0.05 were considered as statistically significant.

## 3 RESULTS

We screened 134 patients for eligibility, of which 18 were excluded since they failed to meet the inclusion criteria. The MTHFR A1298 genotyping analysis on the blood samples of the remaining 116 patients was performed. Of these, 16 patients failed to start treatment (the IVF/ICSI cycles) and PCR‐RFLP results show that these patients had the subsequent MTHFR 1298 genotype: AA (*n* = 8), AC (*n* = 5), and CC (*n* = 3). Statistical analysis of the 100 patients who received controlled ovarian stimulation in the IVF/IESI cycle was performed, and the cycle outcomes were compared according to the patients’ MTHFR 1298 genotypes.

The three genotype groups were compared with respect to basic characteristics, ovarian response, and cycle outcomes (Table 1). No significant differences were found among groups in terms of age, BMI (kg/m^2^), basal FSH and LH levels, type of infertility, infertility duration, AFC, the total dose of gonadotropins used, duration of stimulation, and the total number of oocytes retrieved (Table 1). Women carrying the homozygous CC genotype showed higher levels of AMH than those carrying other genotypes; however, they failed to reach significance. No significant differences were shown among groups in terms of numbers of good‐quality embryos, fertilization rates, clinical pregnancy rates, miscarriage, and live birth rates (Table 1).

**TABLE 1:** Baseline demographic and clinical characteristics as stratified by MTHFR genotypes

Variables	AA (*n* = 34)	AC (*n* = 42)	CC (*n* = 24)	*p*‐value
Age (years)	29.88 ± 4.06	29.19 ± 4.15	30.08 ± 3.67	0.525
BMI (kg/m^2^)	25.73 ± 4.1	26.1 ± 6.41	25.80 ± 4.41	0.964
Basal FSH level (mIU/ml)	5.74 ± 2.3	6.42 ± 2.22	6.23 ± 1.89	0.471
Basal LH level(mIU/ml)	5.14 ± 2.35	4.39 ± 2.25	4.84 ± 2.37	0.274
Basal AMH level (ng/ml)	3.08 ± 1.65	3.32 ± 1.46	4.15 ± 1.67	0.115
Infertility duration	5.45 ± 3.58	4.84 ± 2.98	6.66 ± 5.11	0.178
Type of infertility				
Primary	32(91.2%)	39(92.9%)	23(100%)	0.430
Secondary	3(8.8%)	3(7.1%)	0(0%)	
Azoospermia diagnosis	6(17%)	5(11%)	5(20%)	0.610
AFC	13.45 ± 3.89	12.26 ± 2.56	11.95 ± 2.54	0.186
Type of stimulation protocol				
Agonist	22(61.8%)	33(78.6%)	18(75%)	0.250
Antagonist	13(38.2%)	9(21.4%)	6(25%)	
Total dose of used gonadotropins (75 IU/amp)	1678.50 ± 621.13	1794.04 ± 628.64	1740.62 ± 368.73	0.791
Duration of stimulation (days)	9.8 ± 2.1	10.5 ± 1.6	10.5 ± 1.2	0.212
No. of oocytes retrieved	12.35 ± 8.70	12.28 ± 5.63	13.21 ± 9.20	0.852
No. of MII oocytes	10.64 ± 7.9	11.02 ± 5.2	11.7 ± 7.5	0.750
No. of MI oocytes	0.41 ± 0.70	0.52 ± 0.86	0.43 ± 0.72	0.836
No. of GV oocytes	0.79 ± 1.47	0.35 ± 0.87	0.73 ± 2.0	0.331
No. of good‐quality embryos	4.60 ± 4.51	4.35 ± 2.63	4.86 ± 3.49	0.839
No. of embryos transferred	2.30 ± 0.46	2.34 ± 0.48	2.18 ± 0.39	0.224
No. of cycles not responsive to gonadotropins	2 (5.71)	0 (0)	1 (4.16)	0.334
No. of cycles with no embryos	1 (2.85)	1 (2.38)	1 (4.16)	0.919
No. of all frozen embryo cases	2 (5.71)	3 (7.14)	3 (12.5)	0.719
Fertilization rate without azoospermia cases	0.56 ± 0.24	0.61 ± 0.31	0.64 ± 0.24	0.943
Clinical pregnancy rate/ET	12/30 (40)	15/38 (39.47)	7/19 (36.86)	0.639
Clinical pregnancy rate/ET without including azoospermia cases	11/24 (41.9)	12/33 (48.4)	6/14 (23.1)	0.064
Miscarriage rate/ET	1 (3.33)	1 (0)	2 (5.26)	0.745
Live birth rate /ET	11 (36.66)	14 (36.84)	5 (26.31)	0.098

Note

All quantitative variables are mean ± SD according to the Kruskal‐Wallis and ANOVA tests when appropriate. All qualitative variables are shown as No AFC, antral follicle count; AMH, anti‐Müllerian hormone; BMI, body mass index; ET, embryo transfer; FSH, follicle‐stimulating hormone; GV, germinal vesicle; LH, luteinizing hormone; MCV, mean corpuscular volume; MII, metaphase II.

**FIGURE 1:**
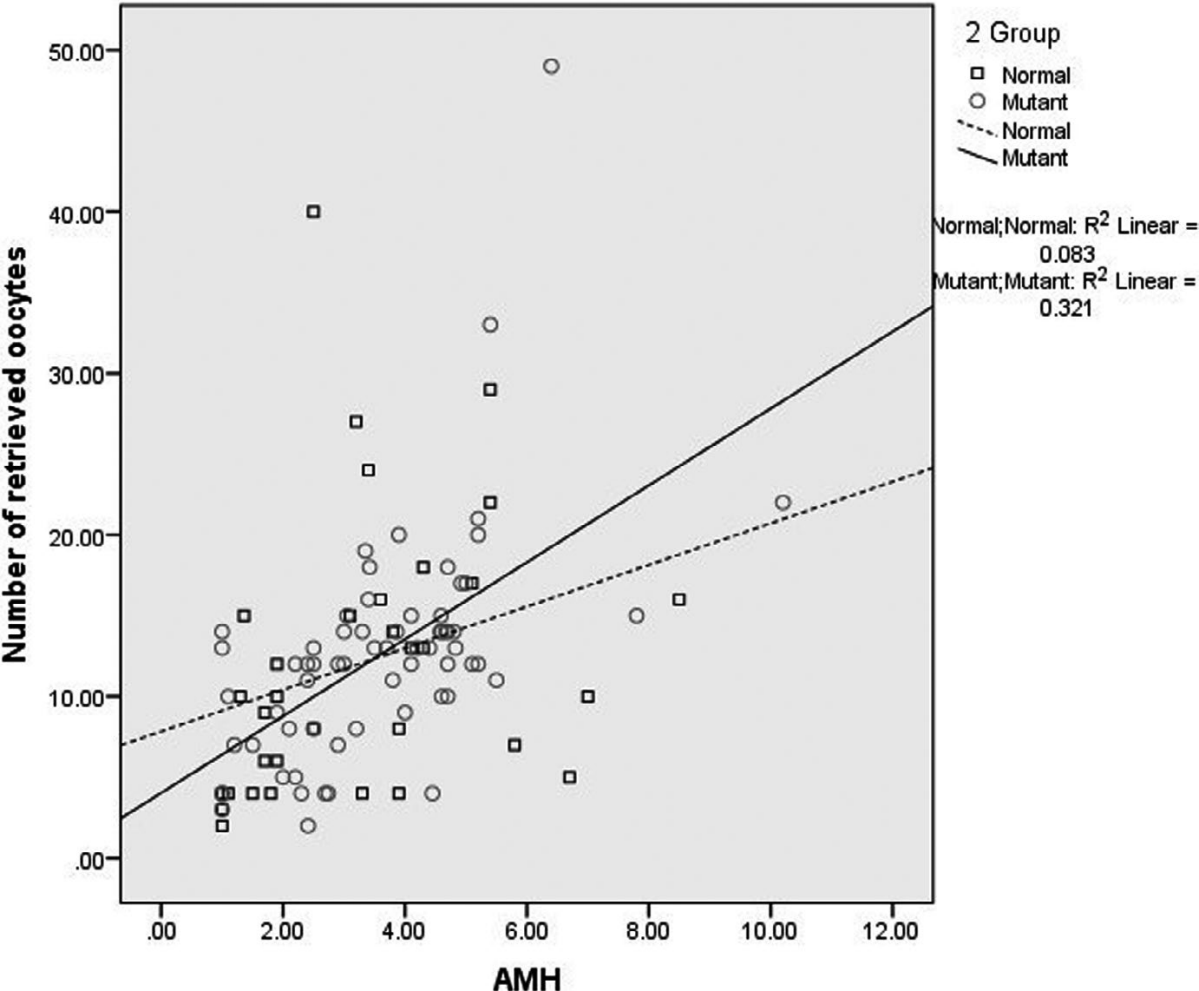
Scatter plot representing the correlation between AMH serum levels and the numbers of oocytes retrieved (NOR) after controlled ovarian stimulation in patients with 34 MTHFR wild genotype (black triangles, black regression line) and 66 mutated MTHFR genotype (gray circle, black dashed regression line) (*r*, Spearman's correlation coefficient)

Linear regression and scatter plot were used to evaluate the associations between AMH concentrations and the number of oocytes retrieved (NOR) separately in the two genotypes (normal and mutant) of MTHFR A1298C (Figure 1). The results showed significant correlations between serum AMH concentrations and NOR in two genotypes. According to the AMH concentrations, there was significantly lower NOR in mutant MTHFR individuals than those with the normal MTHFR genotype. As seen in Figure 1, the mean oocyte numbers for different MTHFR genotype individuals with AMH concentrations ranged from 1 to 5 ng/ml were as follows: 12.09 (normal) and 12.61 (mutant).

Multiple linear regression analysis by the backward manner was used for the detection of significant factors that influenced the numbers of retrieved oocytes. All possible determinants—women's age and BMI, MTHFR genotype (normal and mutated), basal serum FSH, LH levels, adjusted AMH level, AFC, type of stimulation protocol, and total doses of gonadotropins—were entered in the regression analysis. Results demonstrated that serum AMH level had significant positive effects, whereas the FSH levels had a significant negative effect on the total numbers of retrieved oocytes (Table 2), but not a significant difference. Other possible determinants included in the regression model did not have any significant impacts on the numbers of retrieved oocytes (Table 2).

**TABLE 2:** Multiple linear regression analysis by backward manner for detection of possible determinants of the number of oocytes retrieved

Coefficients					
Model	Unstandardized Coefficients		Standardized Coefficients	T	*p*‐value
	ß	Std. Error	ß		
FSH	−0.590	0.315	−0.171	−1.875	0.064
AMH	1.545	0.417	0.359	3.705	0.000
AFC	0.443	0.241	0.179	1.842	0.069
MTHFR (wild and mutated)	0.928	1.484	0.058	0.625	0.534

The multivariable logistic regression test performed in a backward manner was adjusted for age and BMI, then applied to determine the significant predictive variables for live birth in the studied population. All possible important variables such as type and duration of infertility, AFC, serum AMH level, and MTHFR genotype were entered in the model. Our result fails to show any significant predictive when the outcome was live birth (Table 3).

**TABLE 3:** Multivariable logistic regression analysis by the backward manner for determination of the predictive variables for live birth after adjusted for age and BMI (*N* = 100)

Predictive factors	Beta	SE	Wald	Df	Adjusted OR	CI	*p*‐value
Duration of infertility	−1.29	0.068	3.55	1	0.879	0.76–1.00	0.059
AFC	0.144	0.075	3.69	1	1.115	0.99–1.33	0.054

AFC, antral follicle count; BMI, body mass index; CI, confidence interval; OR, odds ratio.

### 3.1 Bioinformatics analysis

The analysis of the MTHFR protein sequence at the UniProt database showed that this protein was expressed as a dimer. Position 429 is in the α‐helix structure; according to the study, this position is not in the domain. The molecular weight and acidic condition of wild‐type protein are higher than mutated protein, but hydrophobicity and isoelectric point of mutated protein are higher. Protein charge in wild‐type protein is −9 and in mutated protein −8. Furthermore, the instability index and aliphatic index of wild protein are less than mutated protein, and the grand average of hydropathicity (GRAVY) was −0.418 and −0.410 for wild and mutated protein, respectively. The difference between wild‐type and mutant form is presented in Table 4.

**TABLE 4:** Difference between wild‐type and mutant forms

Variable	Number of amino acids	Amino acid substitution	Molecular Weight	Acidic (D, E, N, Q)	Basic (K, R, H)	Hydrophobicity	Isoelectric point	The instability index	Charge	Aliphatic index
Wild	656	Glu	74600.16 g/mol	14.48	13.11	40.4	4.97	49.02	−9	80.72
Mutant	656	Ala	74542.12 g/mol	14.33	13.11	40.55	5	48.23	−8	80.87

**FIGURE 2:**
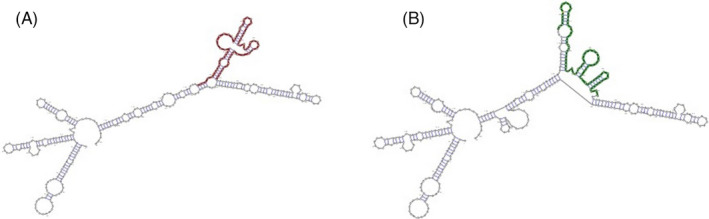
RNAsnp predicts the effects of SNPs on RNA secondary structure. (A) shows wild‐type form, and (B) shows a structural change in the mutant‐type form when the C allele substitutes

Data obtained from SNAP2 database revealed that the Glu429Ala substitutions could affect protein function (prediction: non‐natural; score = 3; expected accuracy: 53%). Moreover, the PolyPhen‐2 server predicted the Glu429Ala substitutions as a benign mutation with a score of 0.021 (sensitivity: 0.95; specificity: 0.80). Data from PROVEAN server showed that this substitution has deleterious effects for MTHFR activity (PROVEAN prediction score: −3.09; SIFT prediction score: 0.100). The effect of A1298C mutation on RNA secondary structure was assessed by RNAsnp server. The data demonstrated substantial changes, a p‐value of less than 0.2, in the secondary structure of mRNA with a distance of 0.3215 (Figure 2).

## 4 DISCUSSION

The present study, for the first time, evaluated the association between the A1298C polymorphism in the MTHFR gene and serum AMH concentrations in women with normal ovarian function. Our findings indicate that there is a trend increase in serum AMH concentrations, albeit not significant, in women with a mutated genotype of MTHFR 1298A>C compared with wild‐type genotype. Interestingly, we found that increasing serum AMH levels in mutated patients is not accompanied by increasing the number of oocyte recovery in these patients. Our study was done in women with a normal ovarian function to exclude the influence of elevated AMH serum concentrations, as seen in polycystic ovary syndrome.

MTHFR is a crucial enzyme in the folate metabolism, which maintains a balance in the flux of folates between DNA synthesis and methylation reactions.^4^ The expression of the folic acid transport protein and MTHFR enzyme in human oocytes and preimplantation embryos enforces the idea for the key role of folate metabolism on aspects of human reproduction.^9^ During times of high folate requirements, such as folliculogenesis and embryogenesis, reduced MTHFR activity due to A1298C polymorphism may impair ovarian function, implantation, and the entire process of pregnancy.^13^, ^14^ Another consequence of reduced MTHFR activity is impaired methionine synthase and homocysteine accumulation, which may induce cytotoxic and oxidative stress and lead to impaired embryo development, and endothelial cells.^18^, ^19^

Despite the relationship between MTHFR polymorphisms and aspects of female reproduction, the effects of the MTHFR polymorphisms on clinical relevance and biochemical parameters are still controversial.^8^, ^9^ In this study, we failed to demonstrate an association between MTHFR genotypes and a number of oocytes retrieved in patients; however, we confirmed previous results that have reported a significant association between AMH levels and the numbers of oocytes retrieved after ovarian stimulation. Rosen et al. in a prospective study of 223 women underwent ART revealed that a MTHFR A1298C polymorphism was associated with higher basal FSH concentrations and lower response to ovarian stimulation, indicating an involvement of the MTHFR A1298C polymorphism in modulating folliculogenesis. However, the absence of a definite effect in the homozygous mutant group may be due to the small number in that genotype.^9^ D'Elia et al. conducted a retrospective study among 82 Brazilian women who underwent IVF treatment for male factor infertility. In their study, patients were divided into two groups according to the presence (*n* = 44) or absence (*n* = 38) of the mutant allele of the 1298 polymorphism. A significant difference was observed regarding the number of oocytes retrieved in women presenting mutant alleles for the *MTHFR* 1298 polymorphism (*p* = 0.044). Interestingly, they found a significant association in terms of good‐quality embryos in women who had a mutant allele.^8^

A few previous studies have evaluated the impact of the MTHFR A1298C polymorphism on IVF cycles with conflicting results.^12^, ^20^ In this study, we found a statistically significant decrease in clinical pregnancy and live birth rates in patients with mutated MTHFR genotype compared to those with the wild genotypes, although the difference was not significant. Haggarty et al. studied 602 women undergoing fertility treatment and observed that women with the homozygous *MTHFR* 1298 AA genotype were significantly linked to a better IVF outcome; however, all patients take folate supplements before attempting to IVF treatment; thus, a possible effect of these polymorphisms could have been masked.^20^ Ensico et al., for the first time, assessed the frequencies of different *MTHFR* alleles in preimplantation embryos. The authors argued that maternal women with 1298A>C genotype had a high chance of achieving clinical pregnancy, suggesting a powerful impact of the 1298C allele on the ability of the embryos to implant. Also, a significant increase in the frequency of CC homozygotes was observed among women undergoing IVF treatment compared with fertile controls.^12^ In contrast to the previously mentioned studies, Patounakis et al that studied 1717 patients attempting their first cycle of IVF reported that there is an association between the MTHFR mutation and the IVF outcomes; thus, they did not recommend this investigation for initial screening.^21^ A study that included 197 women undergoing IVF treatment failed to demonstrate a significant association between A1298C polymorphism and laboratory results including the number and quality of embryos transferred and pregnancy rate.^9^ Also, another study has demonstrated that polymorphism within the MTHFR gene did not seem to help women who underwent fertility treatment to achieve pregnancy.^22^ The relatively small sample size is the limitation of our study, which can produce results with low statistical power and less than expected. In conclusion, our study indicates a statistical difference in serum AMH concentrations in women with mutated MTHFR A1287C genotype compared to those with wild‐type genotype. Elevated AMH levels in mutated patients are an important issue that clinicians need to be aware of to minimize medication errors occurred in treatment strategies. However, the influence of a single polymorphism may be weak, but it may be evident if it coexisted with other mutation. Further studies with large sample size across different ethnic groups are needed to clarify the effect of A1298C MTHFR polymorphisms on female reproduction.

## CONFLICTS OF INTEREST

The authors declare that they have no existing conflicts of interest associated with the manuscript.

## Data Availability

The data that support the findings of this study are available on request from the corresponding author. The data are not publicly available due to privacy or ethical restrictions.
